# Cost of Psychiatric Inpatient Treatment for Dementia in Switzerland: A Case‐Level Analysis of Billing Data

**DOI:** 10.1002/gps.70122

**Published:** 2025-07-04

**Authors:** Elena Bleibtreu, Florian Riese

**Affiliations:** ^1^ Healthy Longevity Center University of Zurich Zurich Switzerland

**Keywords:** alzheimer's disease, billing, cost analysis, dementia, hospital, inpatient, psychiatry, reimbursement, remuneration, Switzerland

## Abstract

**Objective:**

The objective of this study was to investigate the cost of care for patients with a primary diagnosis of dementia in Swiss hospitals under the new TARPSY reimbursement system.

**Methods:**

We used a dataset of the Swiss hospital reimbursement system TARPSY from 2016 to 2019, including all relevant remuneration variables at the patient level, to investigate hospital costs. Costs were analyzed by geographic location and hospital type. Homogeneity coefficients were used to analyze case cost homogeneity.

**Results:**

We identified 7090 cases in the TARPSY database who were treated in Swiss hospitals under the primary diagnosis of dementia from 2016 to 2019. Of these, 6747 cases were included in our analysis. The total case costs and daily costs increased from 2016 to 2019, whereas the length of stay decreased. The average total case cost in 2019 was CHF 34,917 (*σ* = 32,926), corresponding to a daily cost of CHF 946 (*σ* = 373.44). Patients were treated for an average of 39.7 (*σ* = 32.40) days. In 2019, the total costs billed according to TARPSY for 57,939 days of hospital care for dementia as the primary diagnosis were CHF 51.3 million. The case costs differed by region and hospital type. Overall, cost homogeneity for total case cost as a proxy for the quality of the cost calculation was “satisfactory, sufficient” and did not show a clear trend towards improvement during the introduction of TARPSY.

**Discussion:**

Our analysis provides reliable, case‐level cost data for dementia hospital treatment in Switzerland. The total cost of dementia treatment in psychiatric hospitals appears to be much lower than previous estimates had indicated. When correcting for changes in accounting practices, total case costs only increased modestly from 2016 to 2019.

## Summary

- We used case‐level cost data from the Swiss inpatient psychiatric reimbursement system TARPSY to calculate the cost of treatment for dementia as primary diagnosis.
- In 2019, the average total case for dementia as primary diagnosis was CHF 34,917 and the average length of stay of these patients was 39.7 days.
- Total case costs increased only modestly from 2016 to 2019 and appear to be much lower than indicated by previous analysis.

## 1 Introduction

Total healthcare cost can be divided into three main categories: direct cost, indirect cost and intangible cost [1]. Direct cost refers to costs incurred by consumption of healthcare resources (e.g., for diagnosis and treatment) and by consumption of illness‐associated non‐healthcare resources (e.g., for transport, specific diets etc.). Indirect cost denominates costs caused by production loss from morbidity and mortality at all levels (patient, families, society etc.). Intangible cost refers to the subjective burden and loss of quality of life experienced due to an illness and it is rarely included in economic analyses [1, 2]. The provision of healthcare can be classified into either formal care, that is care provided by paid healthcare professionals (thus incurring direct costs) or informal care, that is care provided by unpaid caregivers and communities (leading to indirect costs) [3].

For dementia, the total cost was estimated to be USD 1.3 trillion globally in 2019 [4]. The cost of formal and informal dementia care in the U.S. alone was calculated at USD 450 billion for 2020 [5]. Population ageing, the increasing number of cases and the rise in healthcare expenditures may increase the global total cost of dementia to USD 2.8 trillion by 2030 [4]. For Switzerland, the total annual cost of dementia was estimated at CHF 11.8 billion in 2017 (equivalent to USD 12.6 billion), of which CHF 6.3 billion were direct costs [6]. Consistent with increasing impairment during the later stages of dementia [7], the dementia‐associated cost appears to be particularly high at the end of life [8].

Hospital treatment for dementia is only a minor contributor to total dementia costs: It amounts to approximately 6.4% of the total annual cost in Western Europe according to a recent review and meta‐analysis [9]. However, its absolute cost is still substantial. For Switzerland, the total cost of inpatient hospital care for patients with a primary diagnosis of dementia was estimated at CHF 179.8 million for 2007 [10]. For 2017, these costs were estimated to have risen to CHF 276.0 million [6]. However, these figures were calculated based on assumptions about daily costs and days in hospital derived from the Swiss Hospital Statistics and could not rely on individual case‐level billing data, that is could only provide rough cost estimates.

The data availability for the calculation of dementia care costs in Swiss hospitals recently improved with the implementation of the novel national reimbursement structure for psychiatric inpatient care, TARPSY (“TARif PSYchiatrie”) [11, 12]. Like other diagnosis‐related group (DRG)‐based billing systems, the TARPSY categorizes cases into so‐called psychiatric cost groups (PCGs). To determine the final remuneration for a case, its length of stay (LoS) and the specific cost weight assigned to each PCG are multiplied by the base rate (i.e., the daily base compensation negotiated by the hospital). To discourage longer hospitalizations, daily cost weights decrease with LoS. For example, in 2020 the daily cost weights in the dementia PCG 24A decreased from 1.760 for a LoS of one day to 1.130 for a LoS of 57 days or more.

In 2018, billing according to TARPSY became obligatory for all providers with a public mandate for psychiatric inpatient care in Switzerland, that is, psychiatric hospitals and psychiatric units in other types of hospitals. Starting in 2016, providers were required to collect the respective administrative data and report the billing data. TARPSY data thus includes the exact cost billed for psychiatric inpatient care on a case level. Consequently, TARPSY allows for the exact calculation of psychiatric inpatient care costs for any diagnosis including dementia, as opposed to previous studies that relied on estimates only [6, 10].

The aim of our study was to analyze the costs for inpatient treatment of dementia patients under the new Swiss reimbursement structure TARPSY, that is for treatment in psychiatric hospitals and psychiatric units in other types of hospitals. We furthermore investigated whether there were cost differences by geographical region or hospital type.

## 2 Methods

### 2.1 Dataset and Case Selection

The anonymized TARPSY case‐level billing dataset used in this study was provided by the Swiss Federal Statistical Office (FSO) that maintains a Swiss case cost statistics database (“Bundesamt für Statistik, Fallkostenstatistik 2010–2025”) [13]. The FSO receives validated TARPSY data from SwissDRG AG, a private entity charged with the administration of the system in Switzerland [12]. For validation, SwissDRG AG excludes cases for implausibility or data incompleteness, leading to a lower case number in the TARPSY data than in the Swiss hospital statistic (e.g., 25% fewer in 2019) [14]. Our TARPSY dataset thus includes all administrative data of validated cases billed by providers of inpatient psychiatric care working under the TARPSY V2.0 reimbursement structure, i.e., all psychiatric hospitals and psychiatric units in other types of hospitals that have a public mandate to provide psychiatric services. Specifically, the dataset contains information on primary and secondary diagnoses, procedures (e.g., operations, therapies, etc.), and socioeconomic background (e.g., age, gender, etc.), as well as treatment costs and cost components (e.g., costs for physicians, nursing staff, etc.) at the case level from 2016 to 2019. Even though reimbursement according to the TARPSY reimbursement structure was fully implemented only in 2018, grouping began in 2016. This allows for a comparison between costs in 2016 (i.e., before the implementation of TARPSY) and in 2019 (i.e., after the implementation). All cases with diagnostic codes F00 to F03 and G30 to G31 from the German Modification of International Statistical Classification of Diseases and Related Health Problems (ICD‐10‐GM) as the primary diagnosis were included in our analysis. TARPSY defines the primary diagnosis as the diagnosis that generates most resource consumption during the hospitalization. We included the category G31.2 (“degeneration of nervous system due to alcohol”) in our analysis since alcohol‐related dementia may be categorized here. This category potentially also includes neurodegenerative conditions due to alcohol other than dementia (e.g., cerebellar ataxia). However, we believe it is unlikely that one of those other alcohol‐related neurodegenerative conditions would be a primary diagnosis in psychiatric treatment. We also found that excluding the G31.2 cases from analysis did not meaningfully shift our results because there were only five cases over the 4‐year study period. The calculations for descriptive population characteristics were performed in SPSS 25.

### 2.2 Analysis by Geographical Region and Hospital Type

Following the definition of the FSO, six major geographical regions were considered for the analysis: Lake Geneva (cantons: Vaud, Valais, Geneva—these cantons are French speaking and include the city of Geneva), Espace Mittelland (cantons: Bern, Fribourg, Solothurn, Neuchâtel, Jura—these cantons are partially French and partially German speaking, include the capital of Switzerland (Berne) but also rural, mountainous areas), Northwestern Switzerland (cantons: Basel Stadt, Basel Landschaft, Aargau—these cantons are German‐speaking and include the city of Basel and several commuter towns), Zurich (canton: Zürich—a German speaking canton comprising the metropolitan area of Zurich, Switzerland's most populous city), Eastern Switzerland (cantons: Glarus, Schaffhausen, Appenzell Ausserrhoden, Appenzell Innerrhoden, St. Gallen, Grisons, Thurgau—these cantons are mainly German speaking and rural), and Central Switzerland (cantons: Luzern, Uri, Schwyz, Obwalden, Nidwalden, Zug—these cantons are German speaking and mountainous). Since the dataset included only one case from the seventh major region in Switzerland (Italian‐speaking Ticino), the region was excluded from analysis. Despite differences in language, culture, geography, degree of urbanization etc., all of Switzerland is characterized by a high standard of living and the performance of its healthcare system is among the best among countries in the Organization for Economic Cooperation and Development (OECD) [15].

Hospital types were categorized according to the criteria defined by the FSO [16] in line with the Classification of Health Care Providers (ICHA‐HP) issued by the OECD and the World Health Organization [17]. The classification is based on the annual number of cases treated in a hospital and their distribution across medical specialties with central care hospitals treating more cases in a more varied spectrum of specialties [16]. To improve readability of the results tables, the hospital category labels used by the FSO were replaced by more intuitive abbreviations. GH Uni: K111 = general hospital, central care (university hospital; including psychiatric university hospitals); GH CC: K112 = general hospital, central care; GH PC: K121 = general hospital, primary care (level 3); PSY 1: K211 = psychiatric clinic (level 1); PSY 2: K212 = psychiatric clinic (level 2); GER: K234 = special clinic: geriatrics.

### 2.3 Analysis of Homogeneity

To analyze the homogeneity of case costs we used homogeneity coefficients (HCs). HCs are considered appropriate for investigating cost‐related homogeneity within DRG settings [18]. The HC is the ratio of the mean to the sum of the mean and the standard deviation, *μ*/(*μ* + *σ*). Usually, the HC is used to measure cost homogeneity in DRG groups with the following thresholds: 0–0.5 indicates “poor, insufficient” homogeneity; 0.5–0.599 indicates “satisfactory, sufficient” homogeneity; 0.6–0.649 indicates “good”; and 0.65–1 indicates “very good” homogeneity [18, 19].

## 3 Results

The dataset provided by the FSO contained 7090 cases with a primary diagnosis of dementia spanning the years 2016–2019. Of these, cases with missing data for cantons (41 cases), cases with total case costs equal to zero or with negative cost components (240 cases), and cases with an incorrect psychiatric cost group or other incorrect administrative variables (62 cases) were excluded. The remaining 6747 cases were included in our analysis. The mean age in the case population was 82.31 years (*σ* = 8.484), and 55.1% of cases pertained to female patients.

### 3.1 Total Case Costs, Daily Costs, and Length of Stay

Both total case costs (TCCs) and daily costs (DCs) increased over the study period. The average TCC rose from CHF 31,463 in 2016 to CHF 34,917 in 2019. Correspondingly, the average DC rose from CHF 860 in 2016 to CHF 946 in 2019. The average length of stay (LoS) decreased from 41.8 days in 2016 to 39.7 days in 2019. An overview of the TCCs, DCs, and LoS is given in Table 1. In 2019, the total hospital costs billed according to TARPSY for a total of 57,939 days of hospital care for dementia as the primary diagnosis were CHF 51.3 million.

**TABLE 1:** Case costs and length of stay of dementia patients (2016–2019).

	Year	N	μ	σ	Percentiles		
					25	50	75
Total case costs (CHF)	2016	1770	31,463	22,881.78	15,574	26,591	41,355
	2017	1812	31,608	28,710.13	16,056	26,010	39,913
	2018	1705	33,162	27,847.58	16,092	26,892	41,306
	2019	1460	34,917	32,926.31	16,444	28,109	42,741
Daily costs (CHF)	2016	1770	860	847.16	644	779	922
	2017	1812	832	342.89	662	761	910
	2018	1705	870	380.31	674	796	990
	2019	1460	946	373.44	764	879	1028
Length of stay (days)	2016	1770	41.8	30.92	21	34	55
	2017	1812	41.6	35.88	21	35	53
	2018	1705	42.3	46.98	21	34	52
	2019	1460	39.7	32.40	19	34	51

Abbreviations: CHF: Swiss Francs, N: number of cases, μ: average, *σ*: standard deviation.

### 3.2 Geographic Variation

TCCs increased steadily over the 4 years from 2016 to 2019 in Lake Geneva and Eastern Switzerland. In the major regions Northwestern Switzerland, Zurich, and Central Switzerland, TCCs were falling. Notably, the TCCs in Lake Geneva averaged CHF 44,726, whereas the lowest average TCC was CHF 28,251 in Eastern Switzerland. The average DC of CHF 1027 in Zurich was the highest in Switzerland. In Central Switzerland, DCs have remained rather stable over the 4 years. In Eastern Switzerland and Lake Geneva, DCs have increased over the 4 years. The increase in DCs in Lake Geneva has been continuous and constant and has almost reached the cost level of Zurich in 2019. Notably, length of stay in Zurich was lower than in any other region throughout the study period. Length of stay in the Lake Geneva region, comprising the cities of Geneva and Lausanne, was highest at the beginning of the study period and even increased further over the 4 years. An overview of the share of treated cases, case costs and length of stay by geographic region is given in Table 2.

**TABLE 2:** Share of cases, case costs and length of stay by geographical region in Switzerland.

Panel A: Share of treated cases						
Year	Lake Geneva	Espace midland	Northwestern Switzerland	Zurich	Eastern Switzerland	Central Switzerland
2016	19.49%	27.23%	17.46%	16.95%	13.79%	5.08%
2017	18.10%	31.02%	16.34%	14.24%	16.39%	3.92%
2018	20.88%	30.09%	14.08%	13.55%	15.48%	5.92%
2019	20.96%	27.12%	17.60%	16.64%	17.40%	0.27%
Average	19.79%	28.95%	16.33%	15.30%	15.70%	3.94%

Panel B: Average total case costs (CHF)						
**Year**	**Lake Geneva**	**Espace midland**	**Northwestern Switzerland**	**Zurich**	**Eastern Switzerland**	**Central Switzerland**
2016	36,616	30,789	33,103	30,497	25,719	28,482
2017	42,856	29,660	29,762	27,818	27,848	32,261
2018	46,086	27,224	33,422	31,202	28,834	32,948
2019	53,345	32,899	27,279	27,777	30,602	23,546
Average	44,726	30,143	30,891	29,324	28,251	29,309

Panel C: Average total case costs (CHF)						
**Year**	**Lake Geneva**	**Espace midland**	**Northwestern Switzerland**	**Zurich**	**Eastern Switzerland**	**Central Switzerland**
2016	808	870	805	1026	782	843
2017	918	786	760	996	752	844
2018	953	760	924	993	822	847
2019	1032	879	840	1095	915	890
Average	928	824	832	1027	818	856

Panel D: Average length of stay (days)						
**Year**	**Lake Geneva**	**Espace midland**	**Northwestern Switzerland**	**Zurich**	**Eastern Switzerland**	**Central Switzerland**
2016	49.39	42.37	43.77	33.10	37.80	41.72
2017	49.06	41.81	43.15	30.46	40.54	45.18
2018	52.33	39.77	38.68	32.75	38.96	57.71
2019	52.64	39.82	37.47	29.69	35.70	29.50
Average	50.84	41.01	41.04	31.51	38.35	48.53

Abbreviation: CHF: Swiss Francs.

### 3.3 Variation by Hospital Type

Level 1 psychiatric hospitals treated the most dementia patients (68.6% of the cases on average). General hospitals, primary care level 3 hospitals and special clinics: Geriatrics did not provide any data from 2016 to 2016 to 2018, respectively. Alternatively, the delivered data might have been rejected by SwissDRG AG because of plausibility checks. The most expensive cases were those treated at university hospitals with an associated TCC of CHF 46,877 and a DC of CHF 955. Over the 4 years, both TCCs and DCs have risen continuously and strongly. Average LoS was consistently highest in university hospitals. Over the study period, LoS decreased slightly in the larger psychiatric hospitals that are responsible for most treated cases with dementia as primary diagnosis. An overview of the share of cases, average cost and length of stay by hospital type is given in Table 3.

**TABLE 3:** Share of treated cases, case costs and length of stay by hospital type (2016–2019).

Panel A: Share of treated cases						
	GH Uni	GH CC	GH PC	PSY 1	PSY 2	GER
2016	17.68%	6.03%		73.54%	2.76%	
2017	15.95%	6.40%	4.69%	70.20%	2.76%	
2018	17.36%	7.68%	4.34%	69.33%	1.29%	
2019	17.88%	11.30%	2.60%	61.51%	1.71%	5.00%
Average	17.22%	7.85%	3.88%	68.64%	2.13%	5.00%

**Panel B: Average total case costs (CHF)**						
	**GH Uni**	**GH CC**	**GH PC**	**PSY 1**	**PSY 2**	**GER**
2016	38,068	27,112		30,397	31,802	
2017	43,781	31,852	23,760	29,183	35,713	
2018	49,043	29,610	21,869	30,481	22,673	
2019	56,616	30,191	24,751	30,650	29,729	27,580
Average	46,877	29,691	23,460	30,178	29,979	27,580

Panel C: Average daily costs (CHF)						
	**GH Uni**	**GH CC**	**GH PC**	**PSY 1**	**PSY 2**	**GER**
2016	797	686		888	835	
2017	941	871	753	806	925	
2018	1003	772	714	860	725	
2019	1078	891	735	931	1049	862
Average	955	805	734	871	883	862

Panel D: Average length of stay (days)						
	**GH Uni**	**GH CC**	**GH PC**	**PSY 1**	**PSY 2**	**GER**
2016	51.46	41.10		39.63	44.02	
2017	47.59	47.25	35.34	39.78	45.14	
2018	52.99	42.42	31.26	40.32	35.18	
2019	53.67	36.80	35.76	36.82	32.92	35.41
Average	51.38	41.43	33.90	39.31	41.15	35.41

Abbreviations: GER: specialty clinic: geriatrics; GH CC: general hospital, central care; GH PC: general hospital, primary care (level 3); GH Uni: general hospital, central care (university hospital; including psychiatric university hospitals); PSY 1: psychiatric clinic (level 1); PSY 2: psychiatric clinic (level 2).

### 3.4 Case Cost Homogeneity

The overall total cost homogeneity was in the range of “satisfactory, sufficient”. For DCs, the cost homogeneity was in the range of “good” or “very good”. In the region of Lake Geneva, the homogeneity coefficient increased from 2018 to 2019 for PCG TP24 A (particularly complex dementia cases). However, the homogeneity coefficient in the less resource‐demanding PCG TP24 B shifted to the “poor, insufficient” range in 2019. Some regions, such as Central Switzerland, improved their cost homogeneity from 2018 to 2019, both for TCCs and DCs. An overview of case cost homogeneity is given in Table 4.

**TABLE 4:** Case cost homogeneity coefficients by region and hospital typology (2018 and 2019).

Panel A: Homogeneity coefficients by major region								
Major region	Total case costs				Daily costs			
	TP24 A		TP24 B		TP24 A		TP24 B	
	2018	2019	2018	2019	2018	2019	2018	2019
Lake Geneva	0.55	0.57	0.53	0.43	0.74	0.77	0.70	0.74
Espace midland	0.59	0.63	0.56	0.54	0.72	0.82	0.73	0.76
Northwestern Switzerland	0.66	0.60	0.57	0.57	0.75	0.71	0.80	0.63
Zurich	0.57	0.59	0.62	0.60	0.76	0.70	0.81	0.61
Eastern Switzerland	0.59	0.55	0.52	0.57	0.76	0.74	0.55	0.76
Central Switzerland	0.60	0.66	0.44	0.99	0.57	0.97	0.58	0.78

**Panel B: Homogeneity coefficients by hospital typology**								
**Hospital typology**	**Total case costs**				**Daily costs**			
	**TP24 A**		**TP24 B**		**TP24 A**		**TP24 B**	
	**2018**	**2019**	**2018**	**2019**	**2018**	**2019**	**2018**	**2019**
GH Uni	0.55	0.59	0.54	0.42	0.74	0.79	0.70	0.74
GH CC	0.60	0.59	0.53	0.57	0.68	0.67	0.84	0.81
GH PC	0.70	0.72	0.66	0.64	0.82	0.80	0.83	0.77
PSY 1	0.59	0.59	0.54	0.56	0.72	0.74	0.66	0.67
PSY 2	0.59	0.52	0.64	0.61	0.68	0.65	0.84	0.89
GER		0.59		0.63		0.71		0.70

*Note: N* = 3165; TP24 A and TP24 B refer to the two psychiatric cost groups in which dementia cases are grouped, with TP24 A comprising the more resource intensive cases.

Abbreviations: GER: special clinic: geriatrics; GH CC: general hospital, central care; GH PC: general hospital, primary care (level 3); GH Uni: general hospital, central care (university hospital; including psychiatric university hospitals); PSY 1: psychiatric clinic (level 1); PSY 2: psychiatric clinic (level 2).

## 4 Discussion

We used a case‐level administrative billing dataset to investigate the resource consumption of patients with a primary diagnosis of dementia in Swiss hospitals. The analysis was made possible by the recent implementation of TARPSY, a new reimbursement system for psychiatric inpatient care. Our main findings are that both total case costs and daily costs for dementia hospital treatment increased from 2016 to 2019 whereas the length of stay decreased. The total cost of hospital care for dementia as primary diagnosis in Switzerland revealed by our analysis of psychiatric billing data is much lower than previous estimates. Finally, we find considerable variation of costs between regions and hospital types.

### 4.1 Total Case Costs, Daily Costs and Length of Stay

Both average TCCs and DCs increased over the study period. The average TCCs were CHF 31,463 in 2016 and CHF 34,917 in 2019. A large share of this increase can be explained by a technical detail of TARPSY: Asset utilization costs were newly included under TARPSY but were financed differently before. Accounting for this fact, the increase of the average TCC over the 4‐year period amounted to a modest CHF 774, corresponding to a 2.46% increase (which is only slightly more than the cumulative inflation rate over that period).

The only other comparable case level cost analysis published for Switzerland (that we are aware of) investigated hospitalizations after stroke with or without dysphagia [20]. The average case costs were 27,801 CHF (stroke with dysphagia) and 13,842 CHF (stroke without dysphagia) in 2012, while the LoSs were 14.9 versus 8.9 days. Average TCCs for dementia were much higher, but DCs lower—consistent with the longer treatment durations in a less procedure‐oriented setting. However, the LoS of dementia patients at hospitals decreased, which is in line with previous observations upon the introduction of DRG‐type remuneration systems [21]. From the relatively short period of observation we cannot discern if this decrease in LoS is only the continuation of a historic development towards shorter hospital stays or if it was accelerated (or otherwise impacted) by TARPSY. Analysis of PCGs comprising other major mental illnesses than dementia indeed indicate that the trend towards shorter LoS in Swiss inpatient psychiatry has slowed or dissipated recently [22]. Follow‐up analysis will have to address if this trend continues and if it applies to inpatient care for dementia as well.

### 4.2 Total Cost per Year

To our knowledge, there are two previously published cost estimates for hospital treatment of dementia as the primary diagnosis in Switzerland [6, 10]. For 2007, the total cost was estimated at CHF 179.8 million, of which CHF 109.4 million were incurred in psychiatric clinics for 171,107 treatment days [10]. For 2017, the total cost of hospital care for dementia as a primary diagnosis was estimated to be CHF 276.0 million [6]. Psychiatric hospitals accounted for 71,096 days of care at a total cost of CHF 76.5 million. However, the largest contributor to hospital costs were “central care hospitals”, with CHF 147.5 million. In both previous studies, the estimates of total annual costs for hospitalizations with dementia as the primary diagnosis were much higher than the figure we calculated (CHF 51.3 million for 2019).

We believe that several factors contribute to this large difference. First, the total days spent in hospital care for dementia indeed appear to have decreased drastically (either through a shorter LOS or fewer people being admitted). In 2007, 171,107 days were spent in psychiatric hospitals alone, whereas in 2017, the figure decreased to 71,096. Instead, our analysis of 2019 TARPSY data comprises 57,939 hospital days (including both psychiatric hospitals and psychiatric university hospitals). Second, we believe that both previous studies systematically overestimate costs because of their methodology. Both previous studies did not use case‐level billing data but estimated costs by multiplying hospital days (derived from the Federal hospital statistics) with a daily cost factor specific to a hospital category. For 2017, the authors calculated with a daily cost of CHF 1076 for psychiatric hospitals, whereas our billing dataset revealed much lower daily costs at CHF 806 and CHF 925 for the two types of psychiatric hospitals in our classification in that year. Even more importantly, we believe that in both previous studies the DC assigned to the “central care” hospital category was much too high. For example, for 2017, the authors calculate with a DC of CHF 3268 per hospital day in “central care”, whereas our TARPSY billing data identified a DC of CHF 941.

In our analysis, two methodological factors may have led to a lower calculation: First, our analysis only includes cases billed through TARPSY, which is used in psychiatry only (including psychiatric wards in non‐psychiatric hospitals). Consequently, patients with dementia as the primary diagnosis treated in non‐psychiatric services, for example, neurology, were not included in our analysis. However, we believe that such cases will only contribute a minor fraction of the total hospital days (due to the limited number of beds available in such services). Second, our analysis includes only cases with “plausible” billing data according to SwissDRG AG. This process of data clean‐up by SwissDRG AG is opaque. Compared with the cases in the Swiss hospital statistics (separately registered by the FSO and the basis of the two previous cost estimates), SwissDRG AG considered between 22% and 28% of all case data provided by the FSO for TARPSY (across all diagnostic categories, i. e. not only for dementia as the primary diagnosis) as not plausible for the years 2016–2019 [14]. Extrapolating from the rate of “plausible” cases according to SwissDRG AG in 2019 (75%) to our specific case of dementia as the primary diagnosis would result in a total cost of CHF 68.4 million (CHF 51.3 million/75 × 100)—still much less than the previous cost estimates.

### 4.3 Variation and Homogeneity

TARPSY is DRG‐based and thus aims to define homogeneous groups of treated cases in terms of resource consumption [23]. The variance of the case costs of the treated cases grouped in a PCG should thus be as small as possible. Cost homogeneity within a cost group is also important as cost groups serve as reference units in reimbursement structure development. For hospitals remunerated according to TARPSY, cost homogeneity within one cost group is important for both cost unit accounting and hospital benchmarking. We found the overall total cost homogeneity to be “satisfactory, sufficient”. However, case costs vary both within regions and across hospital types. There are several possible explanations for this. Case costs with the same diagnoses and treatment constellation could have been calculated differently in different regions or hospital types, i. e. there might have been differences in cost accounting. Alternatively, there might be regional wage differences or hospital type‐related capacity holding costs. Similarly, staff time (both nursing and physician) time dedicated per case may differ between settings and could therefore be driving the cost differences. Similarly, different diagnostic and treatment processes may result in different costs. Finally, the variance might also be explained by differences in the case‐mix in different regions or hospital types. Our analysis does not allow us to discern between the different possible causative factors behind the cost variation. However, if the cost variation is due to different allocation approaches in hospitals or regions, this might lead to distortions in the rate calculation and, in the next step, distortions in case remuneration. To remedy such distortions, Swiss healthcare regulators should consider implementing yearly standardized audits of the case cost calculation similar to the mandatory audits of medical coding. Future research should identify additional parameters that are associated with cost variation which would allow further versions of TARPSY to become more accurate.

### 4.4 Strengths and Limitations

Our study has several strengths. First, to our knowledge, it is the first analysis based on a population‐based case‐level billing dataset revealing actual costs billed for hospital dementia treatment. It allowed for analysis by hospital type and geographic region and has the potential to inform the political discourse on the cost of dementia care, shape reimbursement structure development and impact hospital accounting practices. Second, our dataset spans the introduction period of TARPSY, enabling analysis before and after its introduction and thus making its effect more transparent.

Our study also has several limitations. It considers only one type of dementia‐related costs, i. e. hospital costs. Previous studies have shown that the cost of hospital treatment represents only a small fraction of total dementia‐related costs [9]. Furthermore, our analysis only covers hospital costs associated with cases with dementia as a primary diagnosis billed according to TARPSY. Dementia has previously been shown to increase LoS in general hospitals even when occurring as comorbid disorder [24]. Yet, our analysis does not include these additional costs caused by comorbid dementia. Also, our analysis did not differentiate by type of dementia. Evidence from a small sample of dementia patients from London indicates that Lewy body dementia is associated with higher hospital costs than dementia of the Alzheimer's type [25]. Finally, we do not consider the value created by hospitalizations, for example, possible improvements in quality of life or other outcomes meaningful to patients and families.

## 5 Conclusion

Because of population ageing, the number of dementia cases will likely increase drastically over the next decades. This will undoubtedly be associated with an increase in the cost for dementia treatment. However, even in a high‐resource contexts like Switzerland accurate cost data for dementia care is hard to come by. Surprisingly, our cost calculation revealed much lower total costs for dementia hospital care in Switzerland than previous estimates. This finding contradicts the popular defeatist narrative of the “silver tsunami” that is inevitably going to overwhelm our social security systems. Ultimately, only an accurate and detailed understanding of the cost structure will help keep costs manageable.

## Ethics Statement

According to Swiss legislation, research on anonymized datasets such as the one used in our analysis does not require ethics review by a Cantonal Ethics Committee. The data were handled according to the data protection guidelines of the University of St. Gallen.

## Consent

The authors have nothing to report.

## Conflicts of Interest

The authors declare no conflicts of interest.

## Permission to Reproduce Material From Other Sources

The authors have nothing to report.

## Data Availability

The Health Services data used in this study is available from the Federal Statistical Office (FSO) upon reasonable request and following the procedures established by the FSO.
